# Crosscultural adaptation and validation of the simplified Chinese version of the new knee society scoring system

**DOI:** 10.1080/07853890.2025.2564912

**Published:** 2025-10-04

**Authors:** Haodong Wu, Dongping Wan, Yishun Guo, Huanli Bao, Zhao Yang, Shuxin Yao, Chao Xu, Jianbing Ma

**Affiliations:** a Department of Knee Joint Surgery, Honghui Hospital, Xi’an Jiaotong University, Xi’an, China; b Medical College of Yan’an University, Yan’an University, China; c The First Affiliated Hospital, Guangxi University of Chinese Medicine, Nanning, China; d Xi’an medical university, Xi’an, China; e Department of orthopaedics, The First Affiliated Hospital of Air Force Military Medical University, Xi’an, China.

**Keywords:** Chinese, TKA, new-KSS, crosscultural adaptation, validation

## Abstract

**Introduction:**

The 2011 Knee Society Score (new-KSS) describes symptom, satisfaction, expectation, and functional activities in patients undergoing total knee arthroplasty (TKA) and is widely recognized for assessing TKA outcomes. Before using this scale in non-English-speaking populations, it must be translated and culturally adapted to suit the specific demographic.

**Methods:**

The new-KSS was translated and culturally adapted into the new-KSS-CV following international guidelines. Item modifications were informed by the Chinese TKA PROM (CTP), a Delphi expert consensus and cognitive interviews with Chinese TKA patients, aiming to reflect local cultural and activity patterns. The adapted scale was applied in a validation study with preoperative and one-year postoperative assessments. Reliability was assessed using intraclass correlation coefficient (ICC) and Cronbach’s α. Construct validity was tested by comparing the new-KSS-CV with the Chinese versions of the WOMAC and SF-12. Responsiveness was determined by calculating the standardized response means (SRM).

**Results:**

A total of 226 patients were included, with a mean age of 64.7 ± 5.7 years; 142 (62.8%) were female. All patients completed both preoperative and one-year postoperative assessments. Ceiling effects were observed preoperatively in the expectation dimension (40.7% of patients) and postoperatively in symptom (54.4%), functional (72.1%), and standard activities (19.0%) domains. The new-KSS-CV showed good to excellent test–retest reliability (ICC: 0.88–0.97) and internal consistency (Cronbach’s α: 0.74–0.85). The total score correlated significantly and moderately negatively with WOMAC (r = −0.68, *p* < 0.001), while the symptom (r = −0.41), satisfaction (r = −0.42), and functional activities (r = −0.66) domains also showed significant weak to moderate negative correlations (all *p* < 0.001). The expectation domain showed a very weak but significant positive correlation (*r* = 0.19, *p* = 0.005). The functional activities domain and total score of the new-KSS-CV correlated significantly and positively with the SF-12 physical component (*r* = 0.57 and 0.50, respectively; *p* < 0.001). The new-KSS-CV demonstrated a higher SRM (3.89) than WOMAC (–2.41) and SF-12 (1.69 and 0.33).

**Conclusion:**

The new-KSS-CV is a reliable, valid, responsive, and consistent outcome measurement tool that can be used to evaluate the outcome of TKA in Chinese patients.

## Introduction

The Knee Society Score (KSS), developed by the American Knee Society in 1989, was a key tool for quantifying total knee arthroplasty (TKA) outcomes [1]. At that time, TKA primarily targeted sedentary patients, and the scoring system focused on evaluating basic activities like walking and stair climbing, which met the clinical needs of that period [2]. However, as the number of younger TKA patients with longer life expectancies and higher expectations for functional recovery increases, the limitations of the KSS in evaluating complex functional activities have become more apparent [2]. Specifically, the original KSS may not fully capture higher-level functional tasks or patient-reported expectations, and some items may be less relevant across different cultural contexts, potentially limiting its applicability in contemporary clinical and research settings [2].

To meet the growing needs of a new generation of patients, clinical evaluations are gradually shifting from clinician administered measures (CAMs) to patient-reported outcome measures (PROMs), such as the Knee Injury and Osteoarthritis Outcome Score (KOOS), Oxford Knee Score (OKS) and Western Ontario McMaster University Osteoarthritis Index (WOMAC) [3–6]. In 2011, the American Knee Society introduced a revised version of the KSS (new-KSS), which integrates patient-reported outcomes with clinical assessments. This updated system includes patient satisfaction, expectation, and higher-level physical activities to more comprehensive evaluation of functional recovery following TKA [2]. The new-KSS has been validated and culturally adapted in multiple languages, including German, Korean, Spanish, Dutch, and Japanese [7–11].

In China, the increasing prevalence of osteoarthritis (OA) has led to a substantial rise in the number of TKA surgeries [12]. To enable meaningful comparisons of surgical outcomes between Chinese patients and those from other countries, culturally and linguistically appropriate evaluation tools are critical. Although the simplified Chinese new Knee Society Scoring System (SC-NKSS) has been translated and preliminarily validated in China, challenges remain in its practical application [13]. A previous study reported that in 116 assessments, such items were never selected, which may have distorted scores and was attributed to cultural differences between Eastern and Western populations [14]. The developers of the new-KSS emphasized that cultural adaptability is crucial across diverse cultural settings and recommended replacing unsuitable items to better reflect the activity levels and expectations of local patient populations [2]. Given these challenges, optimizing the cross-cultural adaptation of the new-KSS is crucial.

This study took into account the differences in lifestyle and cultural habits between Western and Chinese patients and aimed to improve the Chinese adaptation process of the new-KSS, particularly revising items that were not suitable for Chinese patients. This study aimed to validated the psychometric properties of the revised KSS scale in Chinese patients, including: (1) test-retest reliability, which assessed the consistency of test results over a short period without any intervening treatment or changes; (2) construct validity, by comparing the revised scale with other validated assessment tools, such as WOMAC and SF-12, to evaluate how well it measured the intended construct; and (3) responsiveness, by evaluating the scale’s sensitivity to changes in knee function through comparison of preoperative and postoperative results. Based on these objectives, we hypothesized that the revised KSS scale would demonstrate good reliability, validity, and responsiveness in Chinese patients. The findings of this study may provide a robust tool for evaluating knee function in Chinese clinical settings, offering clinicians an additional instrument for assessing outcomes in patients with OA and supporting improvements in postoperative care and health outcomes in China.

## Patients and methods

### Participants and study design

This study was approved by the Ethics Committee of Xi’an Jiaotong University Affiliated Honghui Hospital. All patients provided written informed consent after being fully briefed on the study’s purpose and procedures. From April to December 2023, patients with end-stage knee OA scheduled for primary unilateral TKA were consecutively recruited. Inclusion criteria were as follows: (1) age ≥18 years with an indication for primary unilateral TKA; (2) cognitive ability to complete the questionnaire; and (3) ability to communicate in Mandarin. Exclusion criteria included: (1) a history of vascular, neurological, or musculoskeletal conditions that could affect physical activity or cause pain; (2) comorbidities severely impairing daily life, such as heart disease, respiratory failure, or mental illness; (3) refusal to participate; and (4) conditions arising during follow-up that could confound the evaluation, such as revision TKA, surgery on the contralateral knee, new onset of hip or lumbar pain, or loss to follow-up.

### Translation and cross-cultural adaptation

The translation and cross-cultural adaptation of the new-KSS were conducted following established guidelines [15,16]. Initially, two native Chinese translators, one an orthopedic surgeon proficient in English and the other a professional translator, independently translated the new-KSS from English to simplified Chinese. This process ensured that both the questionnaire items and scoring instructions remained faithful to the original English version, without any modifications. Afterward, cultural adaptation experts collaborated with the translators to merge the two independent translations into a unified Chinese version. This version was then back-translated into English by two independent translators who were not involved in the initial translation process. Back translation serves as a validation process to confirm that the translated version faithfully represents the content of the original items. This procedure often reveals potential ambiguities or unclear expressions in the translation [17]. An expert review committee composed of orthopedic surgeons, rehabilitation specialists, public health experts, and linguistic professionals evaluated all translation versions. In collaboration with the item pool from the Chinese TKA PROM (CTP) developed by our research team, as well as informed by a Delphi expert consensus and cognitive interviews with Chinese TKA patients, the committee revised and replaced items that were culturally irrelevant to better reflect local cultural norms and activity patterns for Chinese patients [18–20]. The pre-final version of the scoring tool was thus established. To further refine the tool, a pretest was conducted with 30 OA patients, whose feedback was used to revise the questionnaire based on both the back-translated and original English versions. The research team, in collaboration with the expert review committee, reviewed the pretest findings and finalized the simplified Chinese version of the new-KSS (new-KSS-CV). This systematic approach ensured that the new-KSS-CV is both linguistically and culturally adapted.

### Questionnaires

All patients were asked to complete three questionnaires: the new-KSS-CV, WOMAC and SF-12. These questionnaires were self-administered, with a professionally trained research assistant reviewing them for any missing items upon completion. This assistance was intended to reduce the burden on patients, particularly when they needed to answer similar questions across multiple questionnaires. Clinical examinations and radiological assessments were performed by a researcher (CX) trained in knee arthroplasty, following a standardized protocol to ensure consistency across all patients. Data collection was conducted by trained personnel using standardized methods to minimize variation throughout the process. All patients underwent primary TKA at the Knee Joint Department of Xi’an Jiaotong University Affiliated Honghui Hospital, with data gathered at three time points: one week preoperatively (new-KSS-CV, WOMAC and SF-12), one day preoperatively (new-KSS-CV), and one year postoperatively (new-KSS-CV, WOMAC and SF-12).

The new-KSS-CV includes both objective and subjective scores (Supplementary Appendix A) covering six dimensions. The objective score comprises two dimensions: baseline information (10 items, non-scored) and knee indicators (4 examination items, with a maximum score of 75, completed by the doctor). The subjective score encompasses four dimensions: the symptom (3 items, maximum score of 25), the satisfaction (5 items, maximum score of 40), the expectation (3 items, maximum score of 15), and the functional activities (19 items, maximum score of 100, covering subdimensions of functional, standard, advanced and discretionary). Scores for each dimension are obtained by summing the item scores, with dimensions being independent of one another. The main difference between the preoperative and postoperative versions lies in the expectation dimension: the preoperative version assesses the patient’s expectations for treatment, while the postoperative version evaluates whether these expectations were met.

WOMAC is a widely used and validated tool for evaluating patients with OA[5]. The scale includes three dimensions: stiffness (2 items), pain (5 items), and joint function (17 items). Each item is scored from 0 to 4, and the total score is the sum of the three dimensions, ranging from 0 to 96, with higher scores indicating more severe symptom. The Chinese version has been shown to be reliable and valid in Chinese populations [21,22].

The SF-12 is a simplified version of the SF-36 that assesses patients’ self-reported health-related quality of life (HRQoL) through 12 items [23]. The items generate two subscale scores, the physical component summary (PCS-12) and the mental component summary (MCS-12). Each item is weighted according to standard scoring algorithms derived from US general population data, and the subscale scores are norm-based (mean = 50, SD = 10) [24]. Higher scores indicate better health status. Although the original SF-12 was validated in Caucasian populations, its Chinese version has demonstrated good reliability and validity in Chinese populations [25–28].

### Statistical analysis

Kolmogorov–Smirnov tests were employed to assess the normality of the scoring tool scores. Continuous variables were presented as mean ± SD, while categorical variables were reported as frequencies and percentages. All statistical analyses were conducted using SPSS 27.0 software (IBM, New York), with a significance level set at *p* < 0.05.

### Sample size

The sample size of this validation study was determined with reference to published guidelines [17,29]. Recommendations suggest that 30–40 patients are sufficient for pretesting new instruments, while at least 100 patients are needed to adequately evaluate internal consistency, and a minimum of 50 patients is required for assessing floor and ceiling effects as well as construct validity [17,29]. Other guidelines also recommend a ratio of 4 to 10 participants per item, with a total sample size exceeding 100 participants [30,31]. Although no universal consensus exists on the optimal sample size, based on these recommendations the study was designed to recruit approximately 330 participants to ensure sufficient power for psychometric testing [32]. From April to December 2023, a total of 337 patients were enrolled. According to the predefined inclusion and exclusion criteria, 226 patients ultimately completed both preoperative and one-year postoperative assessments, and were included in the final analysis. Since the new-KSS includes distinct preoperative and postoperative versions, both were validated using this same patient cohort.

### Floor and ceiling effects

A scoring tool should accurately reflect the patient’s status using a normal distribution of scores. When scores cluster at the highest or lowest ends, this phenomenon is known as ceiling or floor effects. To evaluate these effects, we analysed the proportion of patients who achieved the highest or lowest scores on each item of the new-KSS-CV. If more than 15% of patients scored at the maximum or minimum for a dimension, this indicated the presence of ceiling or floor effects.

### Reliability

Reliability assessment included evaluations of internal consistency and test-retest reliability. Internal consistency was assessed using Cronbach’s α coefficient. Test-retest reliability reflects the stability and consistency of the scoring tool over time. To evaluate the test-retest reliability of the new-KSS-CV, 50 patients were randomly selected from the 226 participants before surgery and asked to complete the new-KSS-CV again one week later, with no interventions during that period. A one-week interval was chosen based on recommendations suggesting that a range of 2 days to 2 weeks helps avoid changes in patient conditions and recall bias [29,33]. Test–retest reliability was assessed by calculating the intraclass correlation coefficient (ICC; Pearson correlation) and its 95% confidence interval (95% CI). An ICC of ≥0.7 indicates good reliability, while an ICC of >0.8 indicates excellent reliability. Internal consistency was rated as fair (α value of 0.7), good (α value of 0.8), or excellent (α value of 0.9).

### Construct validity

Validity assessment was conducted through construct validity, which refers to the degree to which the scoring tool measures what it is intended to measure. Since there is no gold standard for evaluating TKA outcomes, we used the WOMAC and SF-12, which are widely utilized in Mainland China and have undergone rigorous validation for reliability and validity [21,23]. We compared the scores of the dimensions from all three instruments for 226 patients preoperatively. Pearson correlation coefficients were calculated to assess the correlation between the new KSS-CV dimensions and the WOMAC and SF-12 dimensions. We hypothesized that the correlations would be either less converging or divergent for mental domains (including the expectation dimension) and moderate or strongly converging for physical domains as seen in previous studies [7,9]. The strength of convergent correlations was categorized as weak (0.30–0.50), moderate (0.50–0.70), and strong (>0.70) [34].

### Responsiveness

Responsiveness refers to the ability of a scoring tool to reflect changes in a patient’s status after treatment. Specifically, the difference in scores between preoperative assessments and one-year postoperative assessments of the new-KSS-CV should indicate improvement in the patient’s condition. A larger score difference signifies a stronger capacity of the tool to detect changes [35]. To assess responsiveness, we used the standardized response mean (SRM) to compare preoperative and postoperative scores. SRM values were calculated by dividing the difference in mean scores between preoperative and one-year postoperative assessments by the SD of the scores, where by the 95% CIs were calculated with a jackknife procedure. The goal was to demonstrate the effectiveness of TKA through these outcome measures. An SRM value between 0.2 and 0.5 indicates a small effect, 0.5 to 0.8 indicates a medium effect and greater than 0.8 indicates a large effect. It is anticipated that all dimensions of the new-KSS-CV, except for the expectation dimension, will have an SRM greater than 0.8, as TKA is expected to improve function and alleviate pain in OA patients.

## Results

### Participant demographics

Of the 337 patients who initially completed the new-KSS-CV, 111 were excluded: 1 due to preexisting musculoskeletal disease, 4 due to serious comorbidities, 27 refused participation, 2 underwent revision TKA, 28 had contralateral knee surgery, 12 reported new hip or lumbar pain, and 37 were lost to follow-up. A total of 226 patients were included in the final analysis. Of these, 142 (62.8%) were female. The mean age was 64.7 ± 5.7 years, and the mean body mass index (BMI) was 26.1 ± 3.1 kg/m^2^. Baseline demographic and clinical characteristics are summarized in Table 1.

**Table 1.** Participant demographic data (*n* = 226).

Variable	Value
Age (years; mean ± SD)	64.7 ± 5.7
Sex, number (%)	
Men	84 (37%)
Women	142 (63%)
Side, number (%)	
Right	124 (55%)
Left	102 (45%)
Height (cm, mean ± SD)	160.7 ± 6.7
Weight (kg, mean ± SD)	67.4 ± 9.6
BMI (kg/m^2^; mean ± SD)	26.1 ± 3.1

BMI = body mass index.

### Translation and cross-cultural adaptation

**Figure 1.**
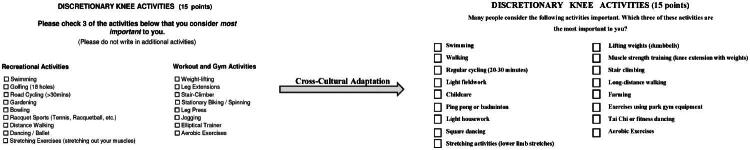
The items of the discretionary knee activities within the new-KSS-CV, which has been culturally adapted from the new-KSS are shown.

During the translation and cross-cultural adaptation process, the expert committee reached a consensus to modify measurement units, converting ‘inches’ to ‘centimetres,’ ‘pounds’ to ‘kilograms,’ and ‘a block’ to ‘100 meters.’ Additionally, the term ‘race’ was replaced with ‘ethnicity’ in the basic information section. Based on the CTP and in consultation with the expert committee, including insights from the Delphi consensus, adjustments were made to the activity subdimension in the new-KSS to ensure cultural relevance [18–20]. For example, original items such as “golf (18 holes), road cycling (30 min), gardening, bowling, tennis (tennis or squash), hiking, dancing/ballet, stretching exercises (yoga), weight lifting, thigh extension machines, stair stepping machines, stationary cycling, leg press machines, jogging, elliptical trainers, and tennis” were replaced with more culturally appropriate items, including ‘walking, regular cycling (20–30 min), light fieldwork, childcare, ping pong or badminton, light housework, square dancing, stretching activities (lower limb stretches), lifting weights (dumbbells), muscle strength training (knee extension with weights), stair climbing, long-distance walking, farming, exercises using park gym equipment, and Tai Chi or fitness dancing’ (Figure 1).

### Floor and ceiling effects

Before surgery, the expectation dimension of the new-KSS-CV exhibited a ceiling effect, with 92 patients (40.7%) expressing very high expectations for postoperative outcomes. One year post-surgery, the symptom, functional and standard activities dimensions also exhibited ceiling effects, with 123 patients (54.4%), 163 patients (72.1%), and 43 patients (19.0%) achieving the highest possible scores in these dimensions, respectively.

### Reliability

The new KSS-CV demonstrated good to excellent reliability across all domains. The ICC ranged from 0.88 to 0.97, indicating excellent reproducibility. Cronbach’s α coefficients for the subscales ranged from 0.74 to 0.85, reflecting high internal consistency (Table 2). All subscales, except for satisfaction (α = 0.88), had ICC values greater than 0.9. Additionally, all subscales, except for the activity domain (α = 0.74), had Cronbach’s α values greater than 0.8.

**Table 2.** Test-retest reliability and internal consistency of the total scores.

Domain	Test 1 (*n* = 226) Mean (SD)	Test 2 (*n* = 50) Mean (SD)	ICC	95% CI	Cronbach’s alpha
Symptom (3 items)/25 points	9.12 (4.37)	8.46 (3.57)	0.95	0.91–0.97	0.85
Satisfaction (5 items)/40 points	14.96 (5.06)	13.20 (4.24)	0.88	0.79–0.93	0.81
Expectation (3 items)/15 points	12.24 (3.13)	12.92 (2.46)	0.97	0.96–0.99	0.85
Total functional activity (19 items)/100 points	39.30 (14.25)	37.78 (13.19)	0.97	0.95–0.98	0.74
Functional activity (5 items)/30 points	13.94 (8.03)	14.82 (7.42)	0.97	0.94–0.98	0.80
Standard activity (6 items)/30 points	13.77 (4.78)	12.56 (4.06)	0.96	0.93–0.98	0.81
Advanced activity (5 items)/25 points	6.42 (3.51)	6.26 (3.65)	0.95	0.91–0.97	0.81
Discretionary activity (3 items)/15 points	5.17 (2.65)	4.14 (3.20)	0.91	0.83–0.95	0.80
Total score/180 points	75.63 (18.66)	72.36 (1783)	0.97	0.95–0.98	0.82

ICC = intraclass correlation coefficient; CI = confidence interval.

### Construct validity

The construct validity of the new-KSS-CV was supported by significant correlations with WOMAC and SF-12 scores. Specifically, new-KSS-CV total and subscale scores (except for the expectation domain) showed moderate to strong negative correlations with WOMAC, while the functional activities domain and total score demonstrated significant positive correlations with the SF-12 physical component. These findings indicate that higher new-KSS-CV scores consistently reflected better clinical outcomes, in line with theoretical expectations (Table 3).

**Table 3.** Construct validity between the new-KSS-CV and the WOMAC.

WOMAC		New-KSS-CV (preoperative form)				
Subscales	Score Mean ± SD	Symptom/25 points	Satisfaction/40 points	Expectation/15 points	Functional activity /100 points	Total score/180 points
Pain/20 points	11.34 ± 3.3	−0.54* (<0.001)	−0.49* (<0.001)	0.31* (<0.001)	−0.35* (<0.001)	−0.47* (<0.001)
Stiffness/8 points	4.20 ± 2.00	−0.46* (<0.001)	−0.37* (<0.001)	0.25* (<0.001)	−0.31* (<0.001)	−0.40* (<0.001)
Function/68 points	35.02 ± 10.33	−0.28* (<0.001)	−0.33* (<0.001)	0.10 (= 0.132)	−0.70* (<0.001)	−0.67* (<0.001)
Total/96 points	50.57 ± 13.68	−0.41* (<0.001)	−0.42* (<0.001)	0.19* (= 0.005)	−0.66* (<0.001)	−0.68* (<0.001)

New-KSS-CV = the simplified Chinese version of the 2011 Knee Society Score; Pearson correlation coefficients (r) when comparing the New-KSS-CV with the WOMAC (p value).

* significant correlation at *p* < 0.05.

The functional activity dimension and total score of the new-KSS-CV exhibited a significant moderate positive correlation with the PCS-12 subscales (*r* = 0.57, *p* < 0.001; *r* = 0.50, *p* < 0.001). In contrast, the correlation between the MCS-12 subscales and the new-KSS-CV dimensions, as well as the total score, was very weak or divergent (Table 4).

**Table 4.** Construct validity between the new-KSS-CV and the SF-12.

SF-12		New-KSS-CV (preoperative form)				
Subscales	Score Mean ± SD	Symptom/25 points	Satisfaction/40 points	Expectation/15 points	Functional activity /100 points	Total score/180 points
PCS-12	33.88 ± 7.73	−0.15* (=0.022)	0.17* (=0.009)	−0.09 (=0.200)	0.57* (<0.001)	0.50* (<0.001)
MCS-12	50.75 ± 7.08	−0.02 (=0.821)	−0.06 (=0.397)	0.10 (= 0.875)	0.01 (=0.928)	−0.005 (=0.935)

New-KSS-CV = the simplified Chinese version of the 2011 Knee Society Score; Pearson correlation coefficients (r) when comparing the New-KSS-CV with the SF-12 (p value).

* significant correlation at *p* < 0.05; PCS = physical component summary; MCS = mental component summary.

### Responsiveness

Responsiveness was assessed using the SRM. Except for the expectation dimension, the SRMs for all other dimensions of the new-KSS-CV exceeded 0.8, indicating strong sensitivity to changes and accurately reflecting improvements after TKA. The new-KSS-CV demonstrated a higher overall SRM (3.89) compared to WOMAC (−2.41) and the SF-12 (PCS = 1.69, MCS = 0.33) (Table 5). The SRM for the symptom dimension was 3.33 for new-KSS-CV, surpassing WOMAC’s pain score of −3.04, while the SRM for functional activity was 3.13, also higher than WOMAC’s score of −2.05. The total SRM for new-KSS-CV was 3.89, compared to −2.41 for WOMAC, further confirming the superiority of new-KSS-CV in capturing clinical improvements post-TKA.

**Table 5.** Responsiveness of the new-KSS-CV compared with the WOMAC and SF-12.

Questionnaire	Mean of change	SD	SRM*(95% CI)
New-KSS-CV			
Symptom (3 items)/25 points	14.91	4.47	3.33 (3.02–3.63)
Satisfaction (5 items)/40 points	13.36	5.42	2.47 (2.26–2.68)
Expectation (3 items)/15 points	−1.10	3.59	−0.31 (−0.45 to −0.16)
Total functional activity (19 items)/100 points	46.81	14.93	3.13 (2.85–3.45)
Functional activity (5 items)/30 points	14.68	8.34	1.76 (1.57–1.96)
Standard activity (6 items)/30 points	13.12	5.30	2.48 (2.23–2.76)
Advanced activity (5 items)/25 points	12.15	4.51	2.69 (2.38–3.00)
Discretionary activity (3 items)/15 points	6.87	2.58	2.66 (2.36–2.97)
Total score(30)/180 points	73.99	19.04	3.89 (3.47–4.28)
WOMAC			
Pain/20 points	−10.53	3.46	−3.04 (−3.36 to −2.71)
Stiffness/8 points	−2.45	2.39	−1.03 (−1.23 to −0.83)
Function/68 points	−25.35	12.38	−2.05 (−2.25 to −1.84)
Total/96 points	−38.32	15.91	−2.41 (−2.65 to −2.16)
SF-12			
PCS-12	14.66	8.69	1.69 (1.45–1.92)
MCS-12	2.18	6.59	0.33 (0.19–0.46)

New-KSS-CV = the simplified Chinese version of the 2011 Knee Society Score; SRM = standardized response mean.

* calculated as the mean change between the preoperative and 12-month scores divided by the SD of the change in score; PCS = physical component summary; MCS = mental component summary; SRM of 0.2–0.5 = small change, 0.5–0.8 = moderate change, and > 0.8 = large change.

## Discussion

The new KSS has been adapted into multiple languages with robust psychometric properties [7–11]. The SC-NKSS, while achieving acceptable results, includes items like golf, bowling, and weightlifting that are rare in Chinese patients’ daily lives, and terms like “leg extension machines” often cause comprehension issues [13]. Moreover, the SC-NKSS may not fully reflect the improvements in patients who received TKA due to the lack of an examination of its responsiveness [13]. To address these issues, our study further optimized the new KSS scale by considering the differences in lifestyle habits and cultural backgrounds between Chinese and Western patients, revising items that are unsuitable for Chinese patients, and validating the psychometric properties of the revised scale among Chinese patients.

Our study results indicate that the revised new-KSS-CV is a valid, reliable, sensitive, and consistent measurement tool for assessing knee function in the Chinese population before and after TKA. Particularly in aspects such as symptom, expectation, satisfaction, and functional activities, the new-KSS-CV provides accurate and reliable assessment outcomes that truly reflect the recovery status of patients after TKA.

Cross-cultural adaptation involves not only language translation but also adjustments that reflect the cultural context of the target country [15,16]. Given the differences in lifestyle and cultural habits between Chinese and Western patients, as well as the adaptability challenges of the original new-KSS in China, our study replaced some items in the scale based on recommendations from an expert committee. Chinese patients are more accustomed to using ‘centimetres’ to describe height, ‘kilograms’ for weight, and ‘metres’ for distance, rather than ‘inches,’ ‘pounds,’ or ‘blocks.’ To minimize confusion caused by unit conversions and better align with the lifestyle habits of Chinese patients, we replaced ‘inches’ with ‘centimetres,’ ‘pounds’ with ‘kilograms,’ and ‘a block’ with ‘100 metres’ in the scale. Additionally, ‘race’ in the basic information was changed to ‘ethnicity,’ which is more culturally appropriate in China.

The study indicates that the discretionary subscale in the original new-KSS functional dimension has poor applicability among Chinese patients, with some items not being selected in actual use [14]. In China, elderly patients typically engage in activities such as walking, playing table tennis, square dancing, and practicing Tai Chi [36]. Sports like golf (18 holes), bowling, tennis, or yoga have a low popularity rate in China and do not match the exercise habits of most patients. Especially in the economically underdeveloped central and western regions, many patients may be unfamiliar with these activities due to their low education levels, affecting their understanding and quality of questionnaire responses [37]. Cultural differences and language barriers may lead to misunderstandings of the original items. For example, they may not be familiar with exercise items such as thigh extenders, step machines, stationary bicycles, leg press machines, and elliptical machines but are very familiar with activities such as climbing stairs, long-distance walking, farming, and using park fitness equipment [38].

During the cross-cultural adaptation process, we utilized the exercise item pool from the CTP, which our research team developed using a combination of qualitative and quantitative research methods, to meet the needs of Chinese patients better [18]. Furthermore, we incorporated patient feedback and expert committee recommendations to adjust and replace items that were unsuitable for Chinese patients [19,20]. We also revised the language of the items multiple times to ensure that patients could accurately understand each one. As a result, patients were able to smoothly comprehend all the items while completing the adjusted questionnaire and provided accurate answers based on the available options. This indicates that the replacement items in the discretionary subscale of the new-KSS-CV are appropriate. These adjustments not only align the exercise items more closely with the lifestyle and cultural habits of Chinese patients but also provide a more accurate reflection of their actual exercise status, thereby validating the strong applicability of the new-KSS-CV among Chinese patients.

The new KSS-CV revealed a ceiling effect in the preoperative expectation dimension, with 92 patients (40.7%) achieving the highest possible scores. This suggests that patients have high expectations for postoperative outcomes, hoping that surgery will completely relieve pain and restore function. This reflects their psychological state, as they desire a thorough improvement in their current situation and ideal therapeutic results from TKA surgery [39]. One year postoperatively, the symptom, functional, and standard activities dimensions also exhibited ceiling effects, with 123 patients (54.4%), 163 patients (72.1%), and 43 patients (19.0%) achieving the highest scores in each dimension. This indicates that more than half of the patients experienced significant recovery after TKA surgery, showing marked improvement in their postoperative health status. Preoperative pain disappeared completely, and patients generally felt that their joint function had returned to normal, enabling them to stand and walk for extended periods without assistive devices and to complete daily activities with ease.

Researchers have demonstrated that many commonly used assessment scales after TKA display notable ceiling effects. For instance, in studies of Western TKA patients, the OKS indicated a ceiling effect of 15.7% one year postoperatively, while the EQ-5D exhibited ceiling effects of 30.2% and 39.8% at six months and one year postoperatively, respectively [40]. Similarly, the new KSS, studied in Western populations, showed a ceiling effect in the walking and standing functional subscales after surgery, and the pain subscale of the KOOS demonstrated a ceiling effect ranging from 15% to 28% [41–43]. Despite the ceiling effects observed in functional and standard activities dimensions, these metrics remain important outcome indicators because they reflect the most common modes of movement for patients. Poor performance in these dimensions suggests that TKA has not effectively improved patient function. Moreover, the ceiling effect, to some extent, highlights the significant efficacy of TKA in patients with end-stage knee OA, as it effectively enhances patient function and quality of life [44].

Moreover, cultural and social background differences between Chinese and Western patients lead to variations in pain expression and medical behaviour [45]. Chinese patients generally tolerate a higher degree of pain and tend to seek medical treatment only when the pain becomes unbearable. Consequently, they often have higher expectations for surgery and are more sensitive to postoperative outcomes [39]. Even minimal improvements after surgery can feel significant to these patients, particularly when contrasted with their preoperative severity.

Therefore, despite the ceiling effect, we believe that the scoring tool remains effective in distinguishing patient status and accurately reflecting the efficacy of TKA surgery. It is crucial to consider the impact of the ceiling effect in future research and clinical assessments. Clinicians should continuously optimize assessment tools by introducing more diverse and refined indicators to fully capture the functional status of patients after surgery. This approach will not only enhance patient prognosis management but also facilitate the development of more effective rehabilitation plans tailored to each patient.

The New-KSS-CV demonstrated exceptional test-retest reliability and good internal consistency across all domains. Reliability refers to the consistency of test results over a short period without any therapeutic changes, while internal consistency indicates the degree of coordination among the various components of the scale. Some researchers suggest that the second test should be conducted within 2 to 21 days to balance the effects of recall bias and changes in disease status [29,33]. However, Terwee and colleagues indicate that the suitability of the selected time period is secondary to the justification provided for that period [29]. In this study, we asked patients to complete two questionnaires within a 1-week interval to assess the scale’s reliability and internal consistency. The results indicated that the New-KSS-CV exhibited excellent test-retest reliability (ICC values ranging from 0.88 to 0.97) and good internal consistency (Cronbach’s α values ranging from 0.74 to 0.85) in all domains. These findings are consistent with translation studies of the 2011 version of the KSS in other languages. For instance, the ICC values for the Japanese, French, and Dutch versions range from 0.65 to 0.88, 0.84 to 0.97, and 0.73 to 0.92, respectively [7,11,46]. Additionally, this study showed Cronbach’s α values between 0.74 and 0.85, which were comparable with other translated versions and the original English version of the new-KSS (with Cronbach’s α ranging from 0.68 to 0.95), all demonstrating good to excellent reliability and internal consistency [2]. These measurement results further confirm the reproducibility of the New-KSS-CV.

No gold standard measure has been established for evaluating the efficacy of TKA [6]. Therefore, this study validated the New-KSS-CV by analysing its correlation with preoperative WOMAC and SF-12 scores, both of which have been validated in China [21,23]. The analysis of convergent validity, based on the validated WOMAC and SF-12 tools, demonstrated that the New-KSS-CV has good construct validity and effectively reflects the knee joint status of patients. The results indicated a significant moderate to strong correlation between the total scores of the New-KSS-CV and the WOMAC (r = −0.68, *p* < 0.001). However, the expectation domain of the New-KSS-CV showed a very weak correlation with the WOMAC scores (*r* = 0.19, *p* = 0.005), which is expected since the WOMAC does not include a domain corresponding to the expectation component of the KSS.

The correlation between the New-KSS-CV and the WOMAC was higher than that with the SF-12, while no significant correlation was observed with the MCS-12 (r = −0.005, *p* = 0.935), which is consistent with expectations. Studies indicate that the physical activity level of current TKA patients has increased compared to the past, with many patients now engaging in activities they were unable to perform preoperatively [47]. Consequently, the activity domain has become an important indicator for post-TKA assessment. The activity domain and total score of the New-KSS-CV showed significant correlations with the PCS-12, with r values of 0.57 (*p* < 0.001) and 0.50 (*p* < 0.001), respectively. Since the SF-12 primarily assesses general health status, and the New-KSS-CV reflects specific knee conditions of TKA patients, correlations—particularly with the MCS-12—were not expected to be strong. This finding is consistent with previous expectations that mental health domains would show weak correlations with New-KSS-CV dimensions [7,9]. The differences in correlation coefficients do not undermine the validity of this study; rather, they highlight the need to further explore the reasons for these differences and assess their potential impact on the scale’s effectiveness.

Responsiveness refers to a scale’s ability to reflect changes in outcomes before and after surgery; the higher the responsiveness, the more sensitive the scale is to detect changes. The study results indicate that the New-KSS-CV has extremely high responsiveness. Among the various dimensions, the total score of the New-KSS-CV showed the most significant change (SRM = 3.89), with the symptom dimension and total functional score also demonstrated substantial changes (SRMs of 3.33 and 3.13, respectively). Compared to the WOMAC (SRM = −2.41) and SF-12 (SRMs of 1.69 and 0.33), the New-KSS-CV exhibited the highest responsiveness. Further analysis revealed that, in comparison to the WOMAC pain dimension (SRM = −3.04) and the PCS-12 (SRM = 1.69), the symptom score of the New-KSS-CV (SRM = 3.33) had greater sensitivity. Additionally, regarding functional scoring, the New-KSS-CV (SRM = 3.13) demonstrated higher responsiveness than both the WOMAC (SRM = −2.05) and the PCS-12 (SRM = 1.69). Since the SF-12 is primarily used to monitor patients’ overall health status and quality of life, it demonstrates weaker responsiveness to post-TKA changes. Therefore, the New-KSS-CV is more sensitive in capturing changes in patient status after TKA compared to other scales, demonstrating strong assessment capabilities.

Despite the positive outcomes of this study, certain limitations exist. First, similar to other validation studies, patients were required to complete three separate scoring tools (new-KSS-CV, WOMAC, and SF-12) simultaneously. This requirement may have increased the burden on respondents, potentially leading to repetitive or missing answers. To mitigate these issues, we arranged for trained research assistants to review the questionnaires as patients filled them out, ensuring that no omissions or invalid responses occurred. However, the involvement of researchers may have resulted in the reliability and validity of the new-KSS-CV being slightly higher in this study than in actual situations where patients complete the questionnaires independently. Second, this study was conducted at a single medical center. Although the center is the largest orthopedic facility in Northwest China, serving both urban and rural patients, the geographical representativeness of the sample may still be insufficient. Future studies should consider multicenter recruitment across the country to reduce biases arising from demographic and cultural differences. Lastly, despite our rigorous translation methods, some inconsistencies may still exist in the language conversion process. Future studies could explore more appropriate vocabulary or phrases and validate them through standardized protocols to ensure the accuracy and consistency of translations.

## Conclusion

The new-KSS-CV is a reliable, valid, responsive, and consistent outcome measurement tool for assessing knee function in Chinese patients both before and after TKA, including patients’ symptoms, expectations, satisfaction, and functional activities. Future studies in larger and more diverse populations may increase the external validity of the new-KSS-CV.

## Supplementary Material

Appendix A.docx

## Data Availability

The findings of this study are supported by data that can be acquired upon request by contacting the corresponding author JBM. Nevertheless, the data cannot be publicly disclosed owing to privacy and ethical considerations.

## Appendix a

Simplified Chinese version of the new-KSS (new-KSS-CV).
