# Supplementary material for: Crosscultural adaptation and validation of the simplified Chinese version of the new knee society scoring system
Source: Ann Med. 2025 Oct 4;57(1):2564912. doi: 10.1080/07853890.2025.2564912 (PMC12498376; doi:10.1080/07853890.2025.2564912)
Supplement: Appendix A.docx [file IANN_A_2564912_SM0057.docx]

**Chinese New Knee Society Score：PRE-OP**

**DEMOGRAPHIC INFORMATION** (To be completed by patient)

1. **Date of Completion 2. Date of Birth** Enter dates as

□□□□/□□/□□ □□□□/□□/□□ yyyy / mm / dd

1. **Height（cm） 4. Weight（kg） 5. Sex**

______ cm ______ kg ○ Male ○ Female

**6. Side of this (symptomatic) knee**

（If both knees will be operated on, please use a different form for each knee）

○Left ○Right

**7. Ethnicity**: ________

**8. Please indicate the expected date and surgeon for your knee replacement operation**

Date □□□□/□□/□□ Surgeon Name: _________

**9. Will this be a primary or revision knee replacement?**

○Primary ○Revision

**To be completed by surgeon**

**10. Charnley Functional Classification (Use Code Below)**  □

A Unilateral Knee Arthritis C1 TKR, but remote arthritis affecting ambulation

B1 Unilateral TKA, opposite knee arthritic C2 TKR, but medical condition affecting ambulation

B2 Bilateral TKA C3 Unilateral or Bilateral TKA with Unilateral or Bilateral THR

**OBJECTIVE KNEE INDICATORS**(To be completed by surgeon)

**ALIGNMENT**

**1．Alignment: measured on AP standing Xray(Anatomic Alignment)**  25 point max

Neutral: 2-10 degrees valgus (25 pts)

Varus: < 2 degrees valgus (-10 pts)

Valgus: > 10 degrees valgus (-10 pts)

**INSTABILITY**

**2. Medial / Lateral Instability: measured in full extension**  15 point max

None (15 pts)

Little or < 5 mm (10 pts)

Moderate or 5 mm (5 pts)

Severe or > 5 mm (0 pts)

1. **Anterior / Posterior Instability: measured at 90 degrees**  10 point max

None (10 pts)

Moderate < 5 mm (5 pts)

Severe > 5 mm (0 pts)

**JOINT MOTION**

1. **Range of motion (1 point for each 5 degrees)**

**Deductions**

**Flexion Contracture**  Minus Points

1-5 degrees (-2 pts)

6-10 degrees (-5 pts)

11-15 degrees (-10 pts)

> 15 degrees (-15 pts)

**Extensor Lag** Minus Points

<10 degrees (-5 pts)

10-20 degrees (-10 pts)

> 20 degrees (-15 pts)

**SYMPTOMS** (To be completed by patient)

1. **Pain level during walking (10 - Score)**

10

9

0

8

7

6

5

4

3

2

1

none severe

1. **Pain with stairs or inclines (10 - Score)**

10

9

8

7

6

5

4

3

2

1

0

none severe

1. **Does this knee feel "normal" to you? (5 points)**

○ Always (5 pts) ○ Sometimes (3 pts) ○ Never (0 pts)

**Total points（25 points）**

**PATIENT SATISFACTION**

1. **Currently, how satisfied are you with the pain level of your knee while sitting? (8 points)**

○Very Satisfied ○Satisfied ○Neutral ○Dissatisfied ○Very Dissatisfied

(8 pts) (6 pts) (4 pts) (2 pts) (0 pts)

1. **Currently, how satisfied are you with the pain level of your knee while lying in bed? (8 points)** ○Very Satisfied ○Satisfied ○Neutral ○Dissatisfied ○Very Dissatisfied

(8 pts) (6 pts) (4 pts) (2 pts) (0 pts)

1. **Currently, how satisfied are you with your knee function while getting out of bed? (8 points)** ○Very Satisfied ○Satisfied ○Neutral ○Dissatisfied ○Very Dissatisfied

(8 pts) (6 pts) (4 pts) (2 pts) (0 pts)

1. **Currently, how satisfied are you with your knee function while (8 points)**

**performing light household duties?**

○Very Satisfied ○Satisfied ○Neutral ○Dissatisfied ○Very Dissatisfied

(8 pts) (6 pts) (4 pts) (2 pts) (0 pts)

1. **Currently, how satisfied are you with your knee function while performing (8 points)**

**leisure recreational activities?**

○Very Satisfied ○Satisfied ○Neutral ○Dissatisfied ○Very Dissatisfied

(8 pts) (6 pts) (4 pts) (2 pts) (0 pts)

**Total points（40 points）**

**PATIENT EXPECTATIONS** (To be completed by patient)

**What do you expect to accomplish with your knee replacement:**

1. **Do you expect your knee joint replacement surgery will relieve your knee pain?**

**(5 points)**

○ no, not at all (1 pt) ○ yes, a little bit (2 pts)

○ yes, somewhat (3 pts) ○ yes, a moderate amount (4 pts)

○ yes, a lot (5 pts)

1. **Do you expect your surgery will help you carry out your normal activities of daily living?**

**(5 points)**

○ no, not at all (1 pt) ○ yes, a little bit (2 pts)

○ yes, somewhat (3 pts) ○ yes, a moderate amount (4 pts)

○ yes, a lot (5 pts)

1. **Do you expect you surgery will help you perform leisure, recreational or sports activities? (5 points)**

○ no, not at all (1 pt) ○ yes, a little bit (2 pts)

○ yes, somewhat (3 pts) ○ yes, a moderate amount (4 pts)

○ yes, a lot (5 pts)

**Total points（15 points）**

**FUNCTIONAL ACTIVITIES** (To be completed by patient)

**WALKING AND STANDING** (30 points)

1. **Can you walk without any aids (such as a cane, crutches or wheelchair)? (0 points)**

○ Yes ○ NO

**2. If no, which of the following aid(s) do you use? (-10 points)**

○ wheelchair (-10 pts) ○ walker (-8 pts) ○ crutches (-8 pts)

○ two canes (-6 pts) ○ one crutch (-4 pts) ○ one cane (-4 pts)

○ knee sleeve / brace (-2 pts) ○ Other ________

**3. Do you use these aid(s) because of your knees? (0 points)**

○ Yes ○ NO

**4. For how long can you stand (with or without aid) before sitting due to knee discomfort?**

(15 points)

○ cannot stand (0 pts) ○ 0-5 minutes (3 pts)

○ 6-15 minutes (6 pts) ○ 16-30 minutes (9 pts)

○ 31-60 minutes (12 pts) ○ more than an hour (15 pts)

**5. For how long can you walk (with or without aid) before stopping due to knee discomfort?**

(15 points)

○ cannot walk (0 pts) ○ 0-5 minutes (3 pts)

○ 6-15 minutes (6 pts) ○ 16-30 minutes (9 pts)

○ 31-60 minutes (12 pts) ○ more than an hour (15 pts)

**Total points（30 points）**

**STANDARD ACTIVITIES** (30 points)

**How much does your knee bother you during each of the following activities?**

1. Walking on an Uneven surface

○**No bother** ○**Slight** ○**Moderate** ○**Severe** ○**Very severe** ○**Cannot do(because of knee)** ○**I never do this**

(5 pts) (4 pts) (3 pts) (2 pts) (1 pts) (0 pts)

2. Turning or pivoting on your leg

○**No bother** ○**Slight** ○**Moderate** ○**Severe** ○**Very severe** ○**Cannot do(because of knee)** ○**I never do this**

(5 pts) (4 pts) (3 pts) (2 pts) (1 pts) (0 pts)

3. Climbing up or down a flight of stairs

○**No bother** ○**Slight** ○**Moderate** ○**Severe** ○**Very severe** ○**Cannot do(because of knee)** ○**I never do this**

(5 pts) (4 pts) (3 pts) (2 pts) (1 pts) (0 pts)

4. Getting up from a low couch or a chair without arms

○**No bother** ○**Slight** ○**Moderate** ○**Severe** ○**Very severe** ○**Cannot do(because of knee)** ○**I never do this**

(5 pts) (4 pts) (3 pts) (2 pts) (1 pts) (0 pts)

5. Getting into or out of a car

○**No bother** ○**Slight** ○**Moderate** ○**Severe** ○**Very severe** ○**Cannot do(because of knee)** ○**I never do this**

(5 pts) (4 pts) (3 pts) (2 pts) (1 pts) (0 pts)

6. Moving laterally(stepping to the side)

○**No bother** ○**Slight** ○**Moderate** ○**Severe** ○**Very severe** ○**Cannot do(because of knee)** ○**I never do this**

(5 pts) (4 pts) (3 pts) (2 pts) (1 pts) (0 pts)

**Total points（30 points）**

**ADVANCED ACTIVITIES** (25 points)

**1. Climbing a ladder or step stool**

○**No bother** ○**Slight** ○**Moderate** ○**Severe** ○**Very severe** ○**Cannot do(because of knee)** ○**I never do this**

(5 pts) (4 pts) (3 pts) (2 pts) (1 pts) (0 pts)

**2. Carrying a shopping bag for 100 meters**

○**No bother** ○**Slight** ○**Moderate** ○**Severe** ○**Very severe** ○**Cannot do(because of knee)** ○**I never do this**

(5 pts) (4 pts) (3 pts) (2 pts) (1 pts) (0 pts)

**3. Squatting**

○**No bother** ○**Slight** ○**Moderate** ○**Severe** ○**Very severe** ○**Cannot do(because of knee)** ○**I never do this**

(5 pts) (4 pts) (3 pts) (2 pts) (1 pts) (0 pts)

**4. Kneeling**

○**No bother** ○**Slight** ○**Moderate** ○**Severe** ○**Very severe** ○**Cannot do(because of knee)** ○**I never do this**

(5 pts) (4 pts) (3 pts) (2 pts) (1 pts) (0 pts)

**5. Running**

○**No bother** ○**Slight** ○**Moderate** ○**Severe** ○**Very severe** ○**Cannot do(because of knee)** ○**I never do this**

(5 pts) (4 pts) (3 pts) (2 pts) (1 pts) (0 pts)

**Total points（25 points）**

**DISCRETIONARY KNEE ACTIVITIES** (15 points)

Many people consider the following activities important. Which three of these activities are

the most important to you?

| Swimming | Lifting weights (dumbbells) |
| --- | --- |
| Walking | Muscle strength training (knee extension with weights) |
| Regular cycling（20-30 minutes） | Stair climbing |
| Light fieldwork | Long-distance walking |
| Childcare | Farming |
| Ping pong or badminton | Exercises using park gym qpuipment |
| Light housework | Tai Chi or fitness dancing |
| Square dancing | Aerobie Exercises |
| Stretching activities (lower limb stretches) |  |

**Please copy all 3 checked activities into the empty boxes below.**

How much does your knee bother you during each of these activities?

**Activity** (Please write the 3 activitesfrom list above)

**1.**_______

○**No bother** ○**Slight** ○**Moderate** ○**Severe** ○**Very severe** ○**Cannot do(because of knee)**

(5 pts) (4 pts) (3 pts) (2 pts) (1 pts) (0 pts)

**2._______**

○**No bother** ○**Slight** ○**Moderate** ○**Severe** ○**Very severe** ○**Cannot do(because of knee)**

(5 pts) (4 pts) (3 pts) (2 pts) (1 pts) (0 pts)

**3._______**

○**No bother** ○**Slight** ○**Moderate** ○**Severe** ○**Very severe** ○**Cannot do(because of knee)**

(5 pts) (4 pts) (3 pts) (2 pts) (1 pts) (0 pts)

**Total points（25 points）**

**Maximum total points (100 points)**

**Chinese New Knee Society Score：POST-OP**

**DEMOGRAPHIC INFORMATION** (To be completed by patient)

1. **Date of Completion 2. Date of Birth** Enter dates as

□□□□/□□/□□ □□□□/□□/□□ yyyy / mm / dd

1. **Height（cm） 4. Weight（kg） 5. Sex**

______ cm ______ kg ○ Male ○ Female

**6. Side of this (surgically treated) knee**

（If both knees have been operated on, please use a different form for each knee）

○Left ○Right

**7. Ethnicity**: ________

**8. Please indicate date and surgeon for your knee replacement operation**

Date □□□□/□□/□□ Surgeon Name: _________

**9. Was this a primary or revision knee replacement?**

○Primary ○Revision

**To be completed by surgeon**

**10. Charnley Functional Classification (Use Code Below)**  □

A Unilateral Knee Arthritis C1 TKR, but remote arthritis affecting ambulation

B1 Unilateral TKA, opposite knee arthritic C2 TKR, but medical condition affecting ambulation

B2 Bilateral TKA C3 Unilateral or Bilateral TKA with Unilateral or Bilateral THR

**OBJECTIVE KNEE INDICATORS**(To be completed by surgeon)

**ALIGNMENT**

**1．Alignment: measured on AP standing Xray(Anatomic Alignment)**  25 point max

Neutral: 2-10 degrees valgus (25 pts)

Varus: < 2 degrees valgus (-10 pts)

Valgus: > 10 degrees valgus (-10 pts)

**INSTABILITY**

**2. Medial / Lateral Instability: measured in full extension**  15 point max

None (15 pts)

Little or < 5 mm (10 pts)

Moderate or 5 mm (5 pts)

Severe or > 5 mm (0 pts)

1. **Anterior / Posterior Instability: measured at 90 degrees**  10 point max

None (10 pts)

Moderate < 5 mm (5 pts)

Severe > 5 mm (0 pts)

**JOINT MOTION**

1. **Range of motion (1 point for each 5 degrees)**

**Deductions**

**Flexion Contracture**  Minus Points

1-5 degrees (-2 pts)

6-10 degrees (-5 pts)

11-15 degrees (-10 pts)

> 15 degrees (-15 pts)

**Extensor Lag** Minus Points

<10 degrees (-5 pts)

10-20 degrees (-10 pts)

> 20 degrees (-15 pts)

**SYMPTOMS** (To be completed by patient)

1. **Pain level during walking (10 - Score)**

10

9

0

8

7

6

5

4

3

2

1

none severe

1. **Pain with stairs or inclines (10 - Score)**

10

9

8

7

6

5

4

3

2

1

0

none severe

1. **Does this knee feel "normal" to you? (5 points)**

○ Always (5 pts) ○ Sometimes (3 pts) ○ Never (0 pts)

**Total points（25 points）**

**PATIENT SATISFACTION**

1. **Currently, how satisfied are you with the pain level of your knee while sitting? (8 points)**

○Very Satisfied ○Satisfied ○Neutral ○Dissatisfied ○Very Dissatisfied

(8 pts) (6 pts) (4 pts) (2 pts) (0 pts)

1. **Currently, how satisfied are you with the pain level of your knee while lying in bed? (8 points)** ○Very Satisfied ○Satisfied ○Neutral ○Dissatisfied ○Very Dissatisfied

(8 pts) (6 pts) (4 pts) (2 pts) (0 pts)

1. **Currently, how satisfied are you with your knee function while getting out of bed? (8 points)** ○Very Satisfied ○Satisfied ○Neutral ○Dissatisfied ○Very Dissatisfied

(8 pts) (6 pts) (4 pts) (2 pts) (0 pts)

1. **Currently, how satisfied are you with your knee function while (8 points)**

**performing light household duties?**

○Very Satisfied ○Satisfied ○Neutral ○Dissatisfied ○Very Dissatisfied

(8 pts) (6 pts) (4 pts) (2 pts) (0 pts)

1. **Currently, how satisfied are you with your knee function while performing (8 points)**

**leisure recreational activities?**

○Very Satisfied ○Satisfied ○Neutral ○Dissatisfied ○Very Dissatisfied

(8 pts) (6 pts) (4 pts) (2 pts) (0 pts)

**Total points（40 points）**

**PATIENT EXPECTATIONS** (To be completed by patient)

**Compared to what you expected before your knee replacement:**

**1. My expectations for pain relief were... (5 points)**

○ Too High- "I'm a lot worse than I thought" (1 pt)

○ Too High- "I'm somewhat worse than I thought" (2 pts)

○ Just Right- "My expectations were met" (3 pts)

○ Too Low- "I'm somewhat better than I thought" (4 pts)

○ Too Low- "I'm a lot better than I thought" (5 pts)

**2. My expectations for being able to do my normal activities of daily living were... (5 points)**

○ Too High- "I'm a lot worse than I thought" (1 pt)

○ Too High- "I'm somewhat worse than I thought" (2 pts)

○ Just Right- "My expectations were met" (3 pts)

○ Too Low- "I'm somewhat better than I thought" (4 pts)

○ Too Low- "I'm a lot better than I thought" (5 pts)

**3. My expectations for being able to do my leisure, recreational or sports activities were... (5 points)**

○ Too High- "I'm a lot worse than I thought" (1 pt)

○ Too High- "I'm somewhat worse than I thought" (2 pts)

○ Just Right- "My expectations were met" (3 pts)

○ Too Low- "I'm somewhat better than I thought" (4 pts)

○ Too Low- "I'm a lot better than I thought" (5 pts)

**Total points（15 points）**

**FUNCTIONAL ACTIVITIES** (To be completed by patient)

**WALKING AND STANDING** (30 points)

1. **Can you walk without any aids (such as a cane, crutches or wheelchair)? (0 points)**

○ Yes ○ NO

**2. If no, which of the following aid(s) do you use? (-10 points)**

○ wheelchair (-10 pts) ○ walker (-8 pts) ○ crutches (-8 pts)

○ two canes (-6 pts) ○ one crutch (-4 pts) ○ one cane (-4 pts)

○ knee sleeve / brace (-2 pts) ○ Other ________

**3. Do you use these aid(s) because of your knees? (0 points)**

○ Yes ○ NO

**4. For how long can you stand (with or without aid) before sitting due to knee discomfort?**

(15 points)

○ cannot stand (0 pts) ○ 0-5 minutes (3 pts)

○ 6-15 minutes (6 pts) ○ 16-30 minutes (9 pts)

○ 31-60 minutes (12 pts) ○ more than an hour (15 pts)

**5. For how long can you walk (with or without aid) before stopping due to knee discomfort?**

(15 points)

○ cannot walk (0 pts) ○ 0-5 minutes (3 pts)

○ 6-15 minutes (6 pts) ○ 16-30 minutes (9 pts)

○ 31-60 minutes (12 pts) ○ more than an hour (15 pts)

**Total points（30 points）**

**STANDARD ACTIVITIES** (30 points)

**How much does your knee bother you during each of the following activities?**

1. Walking on an Uneven surface

○**No bother** ○**Slight** ○**Moderate** ○**Severe** ○**Very severe** ○**Cannot do(because of knee)** ○**I never do this**

(5 pts) (4 pts) (3 pts) (2 pts) (1 pts) (0 pts)

2. Turning or pivoting on your leg

○**No bother** ○**Slight** ○**Moderate** ○**Severe** ○**Very severe** ○**Cannot do(because of knee)** ○**I never do this**

(5 pts) (4 pts) (3 pts) (2 pts) (1 pts) (0 pts)

3. Climbing up or down a flight of stairs

○**No bother** ○**Slight** ○**Moderate** ○**Severe** ○**Very severe** ○**Cannot do(because of knee)** ○**I never do this**

(5 pts) (4 pts) (3 pts) (2 pts) (1 pts) (0 pts)

4. Getting up from a low couch or a chair without arms

○**No bother** ○**Slight** ○**Moderate** ○**Severe** ○**Very severe** ○**Cannot do(because of knee)** ○**I never do this**

(5 pts) (4 pts) (3 pts) (2 pts) (1 pts) (0 pts)

5. Getting into or out of a car

○**No bother** ○**Slight** ○**Moderate** ○**Severe** ○**Very severe** ○**Cannot do(because of knee)** ○**I never do this**

(5 pts) (4 pts) (3 pts) (2 pts) (1 pts) (0 pts)

6. Moving laterally(stepping to the side)

○**No bother** ○**Slight** ○**Moderate** ○**Severe** ○**Very severe** ○**Cannot do(because of knee)** ○**I never do this**

(5 pts) (4 pts) (3 pts) (2 pts) (1 pts) (0 pts)

**Total points（30 points）**

**ADVANCED ACTIVITIES** (25 points)

**1. Climbing a ladder or step stool**

○**No bother** ○**Slight** ○**Moderate** ○**Severe** ○**Very severe** ○**Cannot do(because of knee)** ○**I never do this**

(5 pts) (4 pts) (3 pts) (2 pts) (1 pts) (0 pts)

**2. Carrying a shopping bag for 100 meters**

○**No bother** ○**Slight** ○**Moderate** ○**Severe** ○**Very severe** ○**Cannot do(because of knee)** ○**I never do this**

(5 pts) (4 pts) (3 pts) (2 pts) (1 pts) (0 pts)

**3. Squatting**

○**No bother** ○**Slight** ○**Moderate** ○**Severe** ○**Very severe** ○**Cannot do(because of knee)** ○**I never do this**

(5 pts) (4 pts) (3 pts) (2 pts) (1 pts) (0 pts)

**4. Kneeling**

○**No bother** ○**Slight** ○**Moderate** ○**Severe** ○**Very severe** ○**Cannot do(because of knee)** ○**I never do this**

(5 pts) (4 pts) (3 pts) (2 pts) (1 pts) (0 pts)

**5. Running**

○**No bother** ○**Slight** ○**Moderate** ○**Severe** ○**Very severe** ○**Cannot do(because of knee)** ○**I never do this**

(5 pts) (4 pts) (3 pts) (2 pts) (1 pts) (0 pts)

**Total points（25 points）**

**DISCRETIONARY KNEE ACTIVITIES** (15 points)

Many people consider the following activities important. Which three of these activities are

the most important to you?

| Swimming | Lifting weights (dumbbells) |
| --- | --- |
| Walking | Muscle strength training (knee extension with weights) |
| Regular cycling（20-30 minutes） | Stair climbing |
| Light fieldwork | Long-distance walking |
| Childcare | Farming |
| Ping pong or badminton | Exercises using park gym qpuipment |
| Light housework | Tai Chi or fitness dancing |
| Square dancing | Aerobie Exercises |
| Stretching activities (lower limb stretches) |  |

**Please copy all 3 checked activities into the empty boxes below.**

How much does your knee bother you during each of these activities?

**Activity** (Please write the 3 activitesfrom list above)

**1.**_______

○**No bother** ○**Slight** ○**Moderate** ○**Severe** ○**Very severe** ○**Cannot do(because of knee)**

(5 pts) (4 pts) (3 pts) (2 pts) (1 pts) (0 pts)

**2._______**

○**No bother** ○**Slight** ○**Moderate** ○**Severe** ○**Very severe** ○**Cannot do(because of knee)**

(5 pts) (4 pts) (3 pts) (2 pts) (1 pts) (0 pts)

**3._______**

○**No bother** ○**Slight** ○**Moderate** ○**Severe** ○**Very severe** ○**Cannot do(because of knee)**

(5 pts) (4 pts) (3 pts) (2 pts) (1 pts) (0 pts)

**Total points（25 points）**

**Maximum total points (100 points)**
